# Antioxidant Activity and Acteoside Analysis of *Abeliophyllum distichum*

**DOI:** 10.3390/antiox9111148

**Published:** 2020-11-19

**Authors:** Hak-Dong Lee, Ji Hyun Kim, Qi Qi Pang, Pil-Mun Jung, Eun Ju Cho, Sanghyun Lee

**Affiliations:** 1 Department of Plant Science and Technology, Chung-Ang University, Anseong 17546, Korea; gkrehd1234@naver.com; 2 Department Food Science, Gyeongnam National University of Science and Technology, Jinju 52725, Korea; jihyunkim@gntech.ac.kr; 3 Department Food Science and Nutrition, Pusan National University, Busan 46241, Korea; pangqq@pusan.ac.kr (Q.Q.P.); ejcho@pusan.ac.kr (E.J.C.); 4 Miseonnamu Products Co., Goesan 28035, Korea; kt123@hanmail.net

**Keywords:** *Abeliophyllum distichum*, acteoside, DPPH, hydroxyl radical, HPLC/UV, O_2_^−^ radical

## Abstract

This study determined acteoside and its content in *Abeliophyllum distichum* via HPLC/UV and LC/ESI-MS to obtain insights into the potential use of this plant as an antioxidant agent. Moreover, 1,1-diphenyl-2-picrylhydrazyl (DPPH), hydroxyl (^•^OH), and O_2_^−^ radical scavenging activity assays were performed to assess in vitro antioxidative activity. The DPPH, ^•^OH, and O_2_^−^ radical scavenging activities of *A. distichum* leaf EtOH extracts at a 250 μg/mL concentration were 88.32%, 94.48%, and 14.36%, respectively, whereas those of stem extracts at the same concentration were 88.15%, 88.99%, and 15.36%, respectively. The contents of acteoside in *A. distichum* leaves and stems were 162.11 and 29.68 mg/g, respectively. Acteoside was identified as the main antioxidant compound in *A. distichum* leaves, which resulted in DPPH, ^•^OH, and O_2_^−^ radical scavenging activities of 82.84%, 89.46%, and 30.31%, respectively, at a 25 μg/mL concentration. These results indicate that *A. distichum* leaves and stems containing the antioxidant acteoside can be used as natural ingredients for functional and nutritional supplements.

## 1. Introduction

*Abeliophyllum distichum* Nakai is an important plant resource and represents the only species within the genus Abeliophyllum in the world [1]. *A. distichum* is an endemic plant in Korea and is commonly referred to as white forsythia. Currently, this plant is protected and has been designated as an endangered plant species in Buan-, Goesan-, and Yeongdong-gun, Korea [2,3], with Goesan-gun being the main producer of this resource. Although it has been used as a landscape plant, *A. distichum* has also been found to possess therapeutic value due to its anti-cancer [4], anti-diabetic (via aldose reductase inhibition) [5], and anti-hypertensive properties [3]. *A. distichum* is known to contain some glycosides in its leaves including acteoside, isoacteoside, rutin, and hirsutrin [6], responsible for its anti-inflammatory [7], anti-nociceptive activities [8], and antioxidant activity [6,9,10], and it has also been reported to improve sexual function [11]. Acteoside, a commonly identified phenylpropanoid glycoside in plants [12], is a well-known antioxidant and was first isolated from *Syringa vulgaris* flowers [13]. Moreover, the crude ash contents of *A. distichum* leaves and stems are 1.32% and 0.91%, respectively, and its fructose and glucose contents have been reported as 32.13 and 56.17 mg/g for the leaves, and 11.38 and 10.59 mg/g for the stems, respectively [14].

Free radicals have been linked to the onset of many adverse health effects such as aging, diabetes, cardiovascular, and neurodegenerative diseases [15]. Free radicals including O_2_^−^, hydroxyl (^•^OH), and singlet oxygen (^1^O_2_) are highly reactive in the body and produce reactive oxygen species (ROS) and other free radicals [15]. ROS deteriorate crucial biomolecules for biological functions such as lipids, proteins, DNA, and RNA, thereby leading to cell death and the loss of physiological functions in the body [16]. The 1,1-diphenyl-2-picrylhydrazyl (DPPH) radical is a stable free radical that exhibits a characteristic violet color when reduced by antioxidant materials. Therefore, it is widely used in measuring antioxidant activity [17]. The ^•^OH radical is generated from hydrogen peroxide (H_2_O_2_) and O_2_^−^ by Fenton reaction and is more strongly reactive with biological molecules than other radicals, resulting in many diseases [18]. The O_2_^−^ is generated from molecular oxygen by reduction of one electron in the mitochondrial electron transport chain, endoplasmic reticulum, NADPH oxidase, cytochrome P450, and xanthine oxidase [19]. It is rapidly converted by superoxide dismutase into H_2_O_2_, which becomes a highly reactive ^•^OH radical in the presence of transition metals and peroxynitrite with nitric oxide [20].

ROS, also known as oxygen free radicals by characteristics of unpaired valence electrons or unstable bonds, are among the most important factors that lead to aging and other related diseases such as neurodegenerative diseases [15]. In normal cases, ROS are produced via cellular respiration, metabolic byproducts, enzymatic synthesis, and physical and chemical processes in the body, but they can be removed by the body’s antioxidant system such as enzymatic defenses and antioxidant scavengers [21]. However, the incomplete reduction and overproduction of ROS during various physiological processes may lead to oxidative damage of the cells [22]. Therefore, research on antioxidants that can protect living organisms from ROS oxidation is being actively conducted, with a particular focus on naturally occurring antioxidant agents. Among various naturally occurring antioxidants, phenolic compounds are phytochemicals derived from various plants as a result of their secondary metabolism. In particular, many studies demonstrated that phenolic compounds possess various pharmacological properties including anti-aging, anti-neurodegenerative, and anti-cancer activities, by their antioxidant activity counteracts the effects of ROS in the body [23].

In this study, ethanolic (EtOH) extracts were investigated to determine their acteoside distribution and quantify their content in *A. distichum* via high-performance liquid chromatography (HPLC) coupled with ultraviolet-visible (UV) and electrospray ionization (ESI) ion trap mass spectrometry (MS) detection. Bioactivity of the extracts and acteoside was evaluated by assessing the antioxidant capacity of the *A. distichum* extracts.

## 2. Materials and Methods

### 2.1. Plant Materials and Isolation of Acteoside

**Figure 1:**
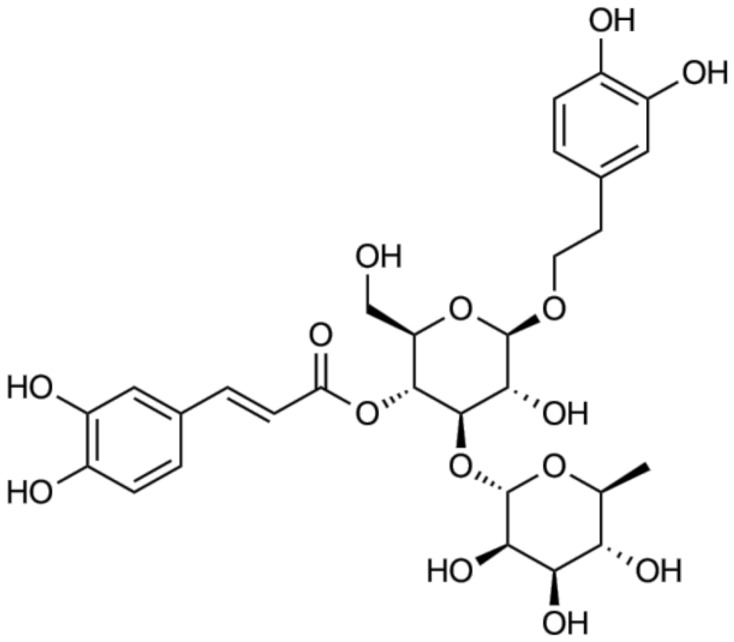
Chemical structure of acteoside.

*A. distichum* leaves and stems were obtained from Miseonnamu Products Co., Goesan, Korea. A voucher specimen (No. LEE19-01) was deposited at the Department of Plant Science and Technology Herbarium, Chung-Ang University, Korea. Dried leaves (300 g) of *A. distichum* were extracted with EtOH for 3 h under reflux and were repeated 3 times. The EtOH extract (120 g) was concentrated, suspended in distilled water, and sequentially partitioned with *n*-hexane (8 g), chloroform (11 g), ethyl acetate (29 g), and *n*-butanol (75 g). A portion of the *n*-butanol fraction (20 g) was subjected to open column chromatography. The column was eluted with a stepwise gradient of chloroform and methanol. The sub-fractions were analyzed by TLC, and the dried residue was further purified with Sephadex LH-20 column chromatography. Fractions of similar composition as determined by TLC were pooled and acteoside (yield, 0.8%) (Figure 1) was obtained by methanol (MeOH) recrystallization. TLC was carried out on pre-coated silica gel 60 F_254_ plates (Merck), developed with chloroform–methanol–water (5.5:4.5:0.2). Open column chromatography was performed on a silica gel column (60–200 mesh, Zeochem) and Sephadex LH-20 (Sigma Aldrich). The purity of acteoside was 98% as determined by HPLC analysis.

### 2.2. Instruments, Chemicals and Reagents

DPPH and 2-deoxy-ribose were purchased from Sigma (St. Louis, MO, USA), FeSO_4_•7H_2_O-EDTA was purchased from Daejung Chemicals and Metals Co. Ltd. (Siheung, Korea), and H_2_O_2_ was obtained from Junsei (Tokyo, Japan). EDTA disodium salt dehydrate was obtained from Samchun Pure Chemical Co. Ltd. (Pyeongtaek, Korea), thiobarbituric acid (TBA) was purchased from Acros Organics (Fair Lawn, NJ, USA), and trichloroacetic acid (TCA) was purchased from Kanto Chemical Co. Inc (Tokyo, Japan). To measure O_2_^−^ radical scavenging activity, Tris was purchased from LPS Solution (Daejeon, Korea), and phenazine methosulfate (PMS), NADH disodium salt, and nitrotetrazolium blue chloride (NBT) were purchased from Bio Basic Co. (Toronto, Canada). Chromatographic analysis was performed using an HPLC system (Agilent 1260 Infinity II Quat Pump, Santa Clara, CA, USA), equipped with a pump, auto-sampler, and diode array detector (DAD WR detector, Arcade, NY, USA). An Ultimate 3000RS system (Thermo Fisher Scientific Inc., Waltham, MA, USA), equipped with an autosampler and PDA-UV detector was used. Mass spectrometric analyses were performed using a Thermo Finnigan LTQ XL ion trap mass spectrometer, with an electrospray ionization (ESI) interface. HPLC grade solvents such as H_2_O, MeOH, and acetonitrile (ACN) were obtained from J. T. Baker (Phillipsburg, PA, USA). Acetic acid (99.7%) was obtained from Samchun Pure Chemicals (Pyeongtaek, Korea).

### 2.3. Preparation of Sample and Standard Solutions for HPLC

The crude EtOH extract (20 mg) of *A. distichum* leaves and stems were dissolved in 1 mL MeOH and filtered using a syringe filter (0.45 μm). A stock solution of the standard compound was prepared by dissolving 1 mg of acteoside in 1 mL MeOH. To construct an acteoside calibration curve, working solutions were prepared by diluting the stock solution to the desired concentrations.

### 2.4. DPPH Radical Scavenging Activity

DPPH radical scavenging activity was measured as described by Hatano et al. [24]. The *A. distichum* EtOH extract and acteoside were first dissolved in EtOH. The samples were then mixed into a 60-μM DPPH solution in 96-well plates, then incubated in the dark at room temperature. After 30 min, absorbance at 540 nm was measured with a microplate reader using l-ascorbic acid as a positive control. DPPH radical scavenging activity was calculated as follows:

DPPH radical scavenging activity (%) = (Abs_c_ − Abs_s_)/Abs_c_ × 100 (1)

Abs_c_: Control absorbance, Abs_s_: Sample absorbance.

### 2.5. •OH Radical Scavenging Activity

^•^OH radical scavenging activity was measured via Fenton reaction [25]. The *A. distichum* EtOH extract and acteoside were dissolved in phosphate-buffered saline, then mixed with 10 mM FeSO_4_^•^7H_2_O-EDTA, 10 mM 2-deoxyribose, and 10 mM H_2_O_2_. The mixtures were incubated at 37 ºC in the dark for 4 h, after which 1% TBA and 2.8% TCA solutions were added, and the mixtures were heated to 100 ºC for 20 min. After cooling, the absorbance was measured at 490 nm using a microplate reader. l-ascorbic acid was used as a positive control. ^•^OH radical scavenging activity was calculated as follows:

^•^OH radical scavenging activity (%) = (Abs_c_ − Abs_s_)/Abs_c_ × 100 (2)

Abs_c_: Control absorbance, Abs_s_: Sample absorbance.

### 2.6. O_2_^−^ Radical Scavenging Activity

O_2_^−^ radical scavenging activity was assessed according to Ewing and Janero et al. [26]. *A. distichum* samples and acteoside diluted in H_2_O were mixed with 0.1 M Tris-HCl (pH 7.4), 100 PMS, 500 NBT, and 500 μM NADH, and incubated at room temperature without light. After 10 min, the absorbance was measured at 560 nm using a microplate reader. l-ascorbic acid was used as a positive control. O_2_^-^ radical scavenging activity was calculated as follows:

O_2_^−^ radical scavenging activity (%) = (Abs_c_ − Abs_s_)/Abs_c_ × 100 (3)

Abs_c_: Control absorbance, Abs_s_: Sample absorbance.

### 2.7. HPLC/UV and LC/ESI-MS Conditions

Quantitative analysis of acteoside was performed using a reverse-phase HPLC system with an INNO C18 column (25 cm × 4.6 mm, 5 μm). The injection volume was 10 μL and was monitored at 330 nm. The column temperature was maintained at 25 °C and the flow rate was set at 0.7 mL/min. A gradient elution system of the mobile phase was composed of 0.5% acetic acid in H_2_O (A) and ACN (B). The elutions were conducted as follows: 90% A at 0 min, followed by 80% A from 0 to 10 min, then 70% A from 10 to 15 min, 50% A from 15 to 20 min, and 0% A from 20 to 30 min, then maintained for 35 min, increased to 90% A from 35 to 40 min, and maintained for 45 min. Regarding the LC/ESI-MS analyses, a Cortecs UPLC T3 column (15 cm × 2.1 mm, 1.6 μm) was used for chromatographic separations. The injection volume was 5 μL and the flow rate was set at 250 μL/min. The mobile phase consisted of 0.1% formic acid in H_2_O (A) and ACN (B). The elutions were conducted as follows: 90% A at 0 min, followed by 80% A from 0 to 10 min, then 70% A from 10 to 15 min, 50% A from 15 to 20 min, 0% A from 20 to 25 min and maintained for 26 min, then increased to 90% A between 26 and 26.5 min and maintained for 30 min. Ionization of analytes was conducted using a negative mode of ESI. The capillary temperature was maintained at 320 °C; the ion source voltage was set at 3.5 kV, and the sheath gas was set at 42 arb. The capillary voltage was set at 10 V in negative mode. The average scan time was 0.01 min while the average time to change polarity was 0.02 min. The collision energy was generally chosen to maintain an approximately 35% abundance of the precursor ion.

### 2.8. Calibration Curve

Calibration curves were constructed by plotting the concentrations of the standard solutions with their respective peak areas. The linearity of the calibration curve was determined based on the correlation coefficient (*r*^2^), after which the acteoside concentrations in the samples were calculated from the calibration curve. The calibration functions were determined based on the peak area *(Y)*, concentration (*X,* mg/mL), and mean values (*n* = 5) ± standard deviation (SD).

### 2.9. Statistical Analysis

All results were reported as the mean ± SD. Statistical significance (*p* < 0.05) was determined via analysis of variance (ANOVA) followed by Duncan’s multiple test using the Statistical Package for the Social Sciences (SPSS, Chicago, IL, USA) program.

## 3. Results and Discussion

The body has natural antioxidant systems such as antioxidant enzymes and antioxidant scavengers [27]. Antioxidant enzymes such as superoxide dismutase, glutathione peroxidases, and catalase have an antioxidant defense by detoxifying ROS [28]. In addition, antioxidant scavengers from dietary origin include tocopherol, ascorbic acid, and polyphenols, which play an important role in ROS detoxification [27]. Therefore, many researchers have focused on the development and identification of natural antioxidant products. A number of studies have reported antioxidant activity of natural products such as flavonoids by measuring their in vitro DPPH, ^•^OH, and O_2_^-^ radical scavenging activity [29,30]. In this study, we identified a flavonoid from *A. distichum* and also evaluated the in vitro antioxidant activities of its extracts as well as its active compound, acteoside.

Table 1 summarizes the DPPH, ^•^OH, and O_2_^-^ radical scavenging activity of the EtOH extracts from *A. distichum* leaves and stems at various concentrations (5–250 μg/mL). *A. distichum* extract treatments increased the DPPH radical scavenging activity in a dose-dependent manner. The leaves and stems of *A. distichum* at a 50 μg/mL concentration exhibited 84.50% and 67.30% activities, respectively. In addition, the IC_50_ values against DPPH of leaves and stems from *A. distichum* were 19.03 and 21.77 μg/mL, respectively, indicating that the leaves of *A. distichum* possessed higher DPPH radical scavenging activity than its stems. Moreover, the ^•^OH radical scavenging activity of *A. distichum* leaves and stems exceeded 80% in all concentrations. Particularly, leaf extracts of *A. distichum* exceeding 50 μg/mL concentrations exhibited ^•^OH radical scavenging activity higher than 90%, which was higher than that of the stem extracts. Previous studies reported that leaf extracts exerted stronger in vitro antioxidant activity than that of stem extracts [29,31]. The O_2_^−^ radical scavenging activity of *A. distichum* leaves and stems was lower than that of the DPPH and ^•^OH radicals. *A. distichum* leaves and stem extracts exhibited O_2_^−^ radical scavenging activity at 100 and 50 μg/mL, respectively.

**Figure 2:**
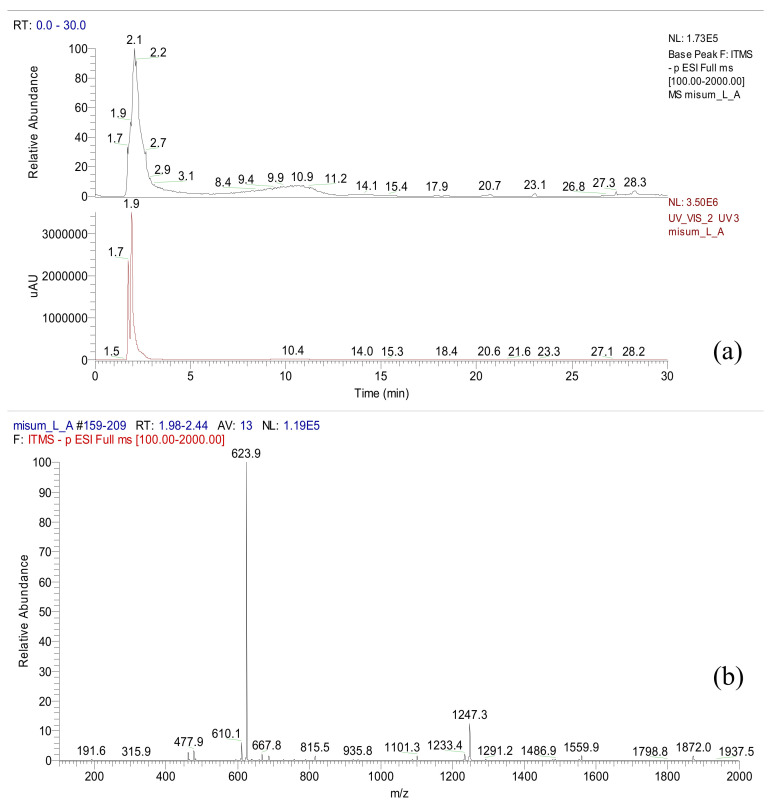
LC/ESI-MS data of *A. distichum* (LC chromatogram (**a**) and ESI-MS data (**b**)).

Acteoside was isolated from the *n*-butanol fraction of *A. distichum*. As depicted in the ^1^H-NMR spectrum, typical patterns of 3,4-dihydroxyphenyl (6.17–7.47 ppm) and rhamnosyl (0.95 ppm) moieties of acteoside were observed (NMR not shown). A total ionization chromatogram in the negative mode revealed the presence of acteoside (*m/z* 624) at a retention time of 2.1 min in *A. distichum* leaves as a major metabolite (Figure 2).

As shown in Table 2, the in vitro antioxidant activity of acteoside derived from *A. distichum* and l-ascorbic acid (1–25 μg/mL) were characterized by measuring their DPPH, ^•^OH, and O_2_^−^ radical scavenging activities. Acteoside significantly increased DPPH radical scavenging activity in a dose-dependent manner. Particularly, an acteoside concentration of 25 μg/mL resulted in a DPPH radical scavenging activity of 82.83%. The IC_50_ values for acteoside and l-ascorbic acid were 4.28 and 0.16 μg/mL, respectively, in DPPH radical scavenging activity. Moreover, the ^•^OH radical scavenging activities for acteoside and l-ascorbic acid at 2.5 μg/mL were 89.46% and 89.95%, respectively. Meanwhile, the IC_50_ values of acteoside and l-ascorbic acid were 0.22 and 0.48 μg/mL, respectively, indicating that *A. distichum*-derived acteoside possessed a strong ^•^OH radical scavenging activity. Furthermore, acteoside from *A. distichum* also dose-dependently enhanced O_2_^−^ radical scavenging activity. Moreover, acteoside and l-ascorbic acid at 25 μg/mL exhibited O_2_^−^ radical scavenging activities of 30.31% and 17.68%, respectively, indicating a strong acteoside-mediated O_2_^−^ radical scavenging activity. A previous study reported that the antioxidant properties of acteoside were likely due to its hydroxyphenylethyl and caffeoyl moieties [32]. It was found to decrease oxidative stress by inhibiting free radicals and lipid peroxidation [30,32]. Furthermore, acteoside attenuated oxidative stress-induced neuronal apoptosis via inhibition of ROS levels and activation of the Nrf2 pathway [33,34]. In previous studies [35,36], compounds such as phenolic glucosides having similar structures to that of acteoside exhibited similar antioxidant activities.

**Figure 3:**
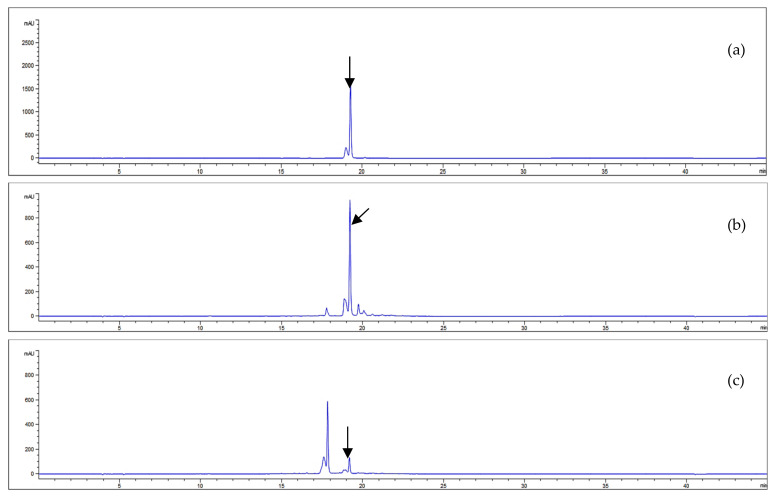
HPLC chromatograms of acteoside (**a**) and the EtOH extracts from *A. distichum* leaves (**b**) and stems (**c**) (330 nm).

Our study investigated the acteoside contents of *A. distichum* leaves and stems via HPLC-UV analysis. Good separations were observed in the HPLC chromatogram with retention time detected at 19.26 min. The HPLC conditions and the results of acteoside quantification are illustrated in Figure 3.

The equation of the standard curve linear calibration was Y = 34,674X − 50,661, where Y represents a given peak area and X is the corresponding acteoside concentration. The analytical method used showed good linearity with a correlation coefficient (*r*^2^) greater than 0.999 (Table 3). The amount of acteoside present in each sample was calculated from the calibration curve. Figure 3 illustrates the chromatographic separation of acteoside and the EtOH extract of *A. distichum* and the results of the quantitative analyses are summarized in Table 3.

Our results revealed that the content of phenolic glycosides in *A. distichum* extracts varied depending on the anatomical structure analyzed. Specifically, the leaf acteoside content (162.11 mg/g) was higher than that in the stems (Table 3). There are similar reports about acteoside content of *A. distichum* from H_2_O extract (171.3 mg/g), 50% prethanol A extract (240.1 mg/g), 70% prethanol A extract (269.4 mg/g), and 100% prethanol A extract (326.1 mg/g) [37]. Moreover, the total phenolic compounds and flavonoid contents of *A. distichum* were 50.64 and 96.47 mg/g in leaves and 13.53 and 18.53 mg/g in stems, respectively [14]. Phenolic glycosides, which belong to a group of natural substances with variable phenolic structure, are found in fruits, bark roots, grains, vegetables, and wine. *A. distichum* contains various active glycosides, such as acteoside, eutigoside B, isoacteoside, rutin, hirsutism, and cornoside [5]. In a previous study, a phenolic glycoside identified as acteoside was found as the main compound present in *A. distichum* leaves [3]. The said compound is reported to possess various biological activities [38]. Our study utilized different assays to measure the antioxidant activities of different parts of *A. distichum.* The design of the study further assessed whether antioxidant activities could vary depending on the plant part containing the active compound.

The previous studies reported the antioxidant activity of *A. distichum*. The callus and flowers of *A. distichum* showed antioxidant activity by radical scavenging activities [39]. In addition, *A. distichum* protected DNA from oxidative stress in the oxidative damage-induced skin fibroblast cells [40]. Furthermore, acteoside isolated from *A. distichum* alleviated oxidative stress-induced cellular damage by decreasing the levels of phosphorylated p53 and γ-H2AX in skin fibroblast cells [41]. *A. distichum* leaves and stems were found to be particularly valuable due to their high content of acteoside, which has therapeutic qualities. Therefore, *A. distichum* could be potentially used as a novel health supplement or in natural medicinal products and antioxidant beverages.

## 4. Conclusions

The antioxidant activity of *A. distichum* EtOH extract and its bioactive compound acteoside were evaluated. Screening of plants containing antioxidants is very important to widen the knowledge of possible sources that can counteract the effects of ROS. This will help prevent the occurrence of ROS-induced diseases such as aging, cancer, and other related diseases. The DPPH, ^•^OH, and O_2_^−^ radical scavenging activities of *A. distichum* leaf EtOH extracts at a 250 μg/mL concentration were 88.32%, 94.48%, and 14.36%, respectively, whereas those of stem extracts at the same concentration were 88.15%, 88.99%, and 15.36%, respectively. The contents of acteoside in *A. distichum* leaves and stems were 162.11 and 29.68 mg/g, respectively. Acteoside was identified as the main antioxidant compound in *A. distichum* leaves, which resulted in DPPH, ^•^OH, and O_2_^−^ radical scavenging activities of 82.84%, 89.46%, and 30.31%, respectively, at a 25 μg/mL concentration. The results in our study demonstrated that *A. distichum* extract has a potent antioxidant activity which can be attributed to its high acteoside content. Moreover, our analyses have shown that the content of phenolic glycosides varies depending on the plant part analyzed. This study established the antioxidant qualities of the leaves and stems of *A. distichium* as an endemic plant to Korea that could be used a basis for developing therapeutic and nutritional products. Therefore, *A. distichum* showed possible use as an effective natural antioxidant for the prevention and treatment of oxidative stress-related diseases such as aging, cardiovascular, and neurodegenerative diseases.

## Figures and Tables

**Table 1:** DPPH, ^•^OH, and O_2_^−^ radical scavenging activities of *A*. *distichum* leaf and stem EtOH extracts.

Treatment (μg/mL)	DPPH (%)		^•^OH (%)		O_2_^−^ (%)	
	Leaves	Stems	Leaves	Stems	Leaves	Stems
5	15.40 ± 2.74 ^e^	23.30 ± 2.26 ^f^	83.84 ± 0.38 ^d^	82.83 ± 0.31 ^d^	-	-
10	35.86 ± 3.81 ^d^	34.37 ± 3.51 ^e^	87.47 ± 0.42 ^c^	87.21± 0.32 ^c^	-	-
25	75.29 ± 3.31 ^c^	61.74 ± 1.25 ^d^	89.79 ± 0.47 ^b^	88.52 ± 0.46 ^b^	-	-
50	84.50 ± 0.97 ^b^	67.30 ± 1.03 ^c^	90.12 ± 0.18 ^b^	89.39 ± 0.49 ^a^	-	0.93 ± 3.83 ^c^
100	81.71 ± 1.51 ^b^	71.76 ± 1.38 ^b^	90.13 ± 1.10 ^b^	89.62 ± 1.34 ^a^	9.89 ± 0.98 ^b^	10.46 ± 2.02 ^b^
250	88.32 ± 2.18 ^a^	88.15 ± 3.56 ^a^	94.48 ± 0.54 ^a^	88.99 ± 0.26 ^a,b^	14.36 ± 0.97 ^a^	15.36 ± 1.99 ^a^

The values represent the mean ± SD. Different letters (^a–f^) indicate significant differences (*p* < 0.05), as determined by Duncan’s multiple range test.

**Table 2:** DPPH, ^•^OH, and O_2_^−^ radical scavenging activities of *A. distichum*-derived acteoside.

Treatment (μg/mL)	DPPH (%)		^•^OH (%)		O_2_^−^ (%)	
	Acteoside	l-Ascorbic Acid	Acteoside	l-Ascorbic Acid	Acteoside	l-Ascorbic Acid
1	15.60 ± 2.00 ^e^	70.04 ± 3.40 ^c^	62.40 ± 0.84 ^e^	56.92 ± 1.31 ^e^	3.60 ± 0.18 ^d^	6.63 ± 1.82 ^c^
2.5	33.12 ± 2.19 ^d^	82.94 ± 3.60 ^b^	80.41 ± 0.27 ^d^	68.94 ± 0.95 ^d^	9.44 ± 0.95 ^c^	6.66± 2.24 ^c^
5	59.90 ± 1.76 ^c^	94.15 ± 2.73 ^a^	86.08 ± 0.11 ^c^	73.29 ± 1.67 ^c^	9.89 ± 0.38 ^c^	7.20 ± 1.76 ^c^
10	76.25 ± 3.32 ^b^	93.63 ± 2.14 ^a^	87.52 ± 0.27 ^b^	80.30 ± 1.05 ^b^	15.71 ± 0.61 ^b^	10.43 ± 2.80 ^b^
25	82.84 ± 1.65 ^a^	94.74 ± 3.60 ^a^	89.46 ± 0.85 ^a^	89.95 ± 0.47 ^a^	30.31 ± 0.34 ^a^	17.68 ± 3.62 ^a^

The values represent the mean ± SD. Different letters (^a–e^) indicate significant differences (*p* < 0.05), as determined by Duncan’s multiple range test. L-Ascorbic acid was used as a positive control.

**Table 3:** Calibration curve and content of acteoside.

Compound	t_R_^a^	Calibration Equation ^b^	Correlation Factor, *r*^2 c^	Content (mg/g Extract)	
				Leaves	Stems
Acteoside	19.26	Y = 34,674X − 50,661	0.999	162.11 ± 0.63	29.68 ± 0.60

^a^ t_R_ = retention time.^b^ Y = peak area, X = concentration of standard (mg/mL).^c^ *r*^2^ = correlation coefficient for three data points in the calibration curve.
